# Experience of Elderly People Regarding the Effect of Yoga/Light Exercise on Sedentary Behavior: A Longitudinal Qualitative Study in Madhya Pradesh, India

**DOI:** 10.3390/geriatrics5040103

**Published:** 2020-12-11

**Authors:** Priyanka Gour, Anita Choudhary, Krushna Chandra Sahoo, Maria Jirwe, Mats Hallgren, Vinod Kumar Diwan, Vijay K. Mahadik, Vishal Diwan

**Affiliations:** 1 Department of Physiology, R D Gardi Medical College, Ujjain 456001, India; priyankardgmc@gmail.com (P.G.); dranitats@gmail.com (A.C.); uctharc@sancharnet.in (V.K.M.); 2 ICMR-Regional Medical Research Centre, Bhubaneswar 751023, India; sahookrushna79@gmail.com; 3 Department of Neurobiology, Care Sciences and Society, Karolinska Institutet, 11428 Stockholm, Sweden; maria.Jirwe@ki.se; 4 Department of Health Sciences, Red Cross University College, 14122 Huddinge, Sweden; 5 Department of Global Public Health, Karolinska Institutet, 17177 Stockholm, Sweden; mats.hallgren@ki.se (M.H.); vinod.diwan@ki.se (V.K.D.); 6 Division of Environmental Monitoring and Exposure Assessment (Water & Soil), ICMR-National Institute for Research in Environmental Health, Bhopal 462030, India

**Keywords:** older adults, physical activity, yoga, light exercise, intervention, aging, India

## Abstract

This study is set on the background of a randomized control trial (RCT) in which intervention was carried to observe the effects of yoga/light exercise on the improvement in health and well-being among the elderly population. A longitudinal qualitative study was conducted as part of RCT interventions to explore the experience of the elderly practicing yoga/light exercise in relation to sedentary behavior in the Ujjain district of Madhya Pradesh, India. Participants of the RCT were selected for this study. Eighteen focus group discussions were conducted—six during each phase of RCT interventions (before, during, and after). The findings regarding motivating and demotivating factors in various phases of intervention were presented in three categories: experience and perception of the effects of yoga/light exercise on sedentary behavior (1) before, (2) during, and (3) after intervention. This study explores the positive effect of yoga/light exercise on sedentary behavior and subjective well-being on the elderly population. They were recognized to have undergone changes in their physical and emotional well-being by consistently practicing yoga/light exercise. The main driving factors were periodic health check-ups and the encouragement of qualified trainers without any cost. This study concludes with the notion that these interventions should be encouraged in the community to use physical exercise as a method to better control the physical and social effects of aging.

## 1. Introduction

India, with the world’s second-largest population, has undergone a dramatic demographic transition over the last decades; the population of older adults has almost tripled [1]. The percentage of older adults in the Indian population has been increasing in recent years and, this increase is likely to continue. The number of elderly adults in India is projected to reach 158.7 million by 2025 [2]. According to the World Health Organization (WHO), India’s current 60 million elderly population is projected to exceed 227 million by 2050, an increase of nearly 280 percent compared to the current year. This transition will place additional burdens on society and the economy of the country, as a large part of the population will be confronted with geriatric challenges [3,4]. In addition, rapid urbanization and modernization of society are responsible for the breakdown of family values and the scaffolding of family support, social isolation, economic insecurity, and the abuse of the elderly which, in turn, lead to a higher risk of psychological illness and a higher need for care for the elderly [5,6,7,8].

In South Asia, deaths from non-communicable diseases (NCDs) will increase from 51% to 72% by 2030, and around 30% of these deaths are preventable. In addition, low-income countries are still struggling with communicable diseases [9]. According to the WHO, sedentary lifestyles increase all causes of death; double the risk of obesity, cardiovascular disease, and diabetes; and increase the risk of high blood pressure, colon cancer, osteoporosis, depression and anxiety, lipid disorders, etc. [10]. Elderly people in India are thus more likely to suffer from chronic illness than from acute illness, as well as NCDs—primarily cardiovascular, metabolic, and degenerative disorders. Moreover, communicable diseases are also on the rise among the elderly [11]. A previous study in India found that almost 98% of respondents felt that their everyday lives had been affected by old age. Of these, 86% felt that their daily activities were partially affected by age, half of them felt neglected by their families, 47% felt unhappy in their lives, and 36% felt burdened by their families [12]. This indicates that aging is an inevitable phase of life, associated with many unpleasant physical, psychological, and social challenges [5,6,7,8].

Although aging is an irreversible and inescapable phase of life, physical activity has proved to be a useful tool in tackling age-related problems. Current trends in the study of physical activity and inactivity therefore deserve close attention. As per previous literature, yoga and meditation can be conceptualized as a family of nuanced emotional and careful regulatory activities, in which mental and associated somatic events are influenced by a particular collection of focus. Many recent behavioral, electroencephalographic, and neuroimaging studies have revealed the significance of studying meditation-related states and traits to improve understanding of cognitive and affective neuroplasticity, concentration, and self-awareness, as well as their potential clinical consequences. There is mounting evidence to suggest that there is a potential health risk threshold related to the degree of activity or inactivity [13]. Older adults who were physically active experienced a later onset of chronic disease compared to their sedentary counterparts, particularly diabetes and obesity [14]. The hyper-inflammatory state is an inexorable upshot of advanced age. Exercise training and increased physical activity are shown to be effective counter-inflammatory measures against hyper-inflammation [15]. A systematic review supported the relationship between sedentary behavior and mortality in older adults in the study [16]. In a study on the elderly from Italy, it was suggested that exercise and dietary interventions may promote mental health throughout an individual’s lifetime [17]. However, understanding of physical activity in elderly people has not been greatly explored in low–middle-income countries (LMICs), which may differ from those in high-income countries. This is a fact that must be considered before any such intervention is launched in a country like India. This study explored the perspective of the elderly about the feasibility of yoga/light exercise practice and its effect on well-being and the prevention of sedentary behavior.

## 2. Materials and Methods

### 2.1. Study Design and Setting

A longitudinal qualitative study was conducted as part of randomized control trial (RCT) interventions in Ujjain district of Madhya Pradesh, India. We used qualitative methods to understand the phenomenon thoroughly and in detail, which uses inductive techniques to create new insights into phenomena that are difficult to quantify. Longitudinal qualitative research (LQR) primarily centers on experience interpretation over time, with the key topic of interpretation being transformation, including knowing what improvements have occurred with respect to certain phenomena, assessments, or interventions, or embedded as part of a randomized controlled trial. The LQR has been used for two purposes: to gather data about a phenomenon over two or more periods of time, and to evaluate data comparisons over time [18].

The Ujjain district has a geographical area of nine thousand square kilometers and has a population of about 73 million, of which about nine percent are senior citizens. The RCT was conducted for a 12-week period consisting of 60-min sessions; yoga/light exercise intervention was performed three times a week. A total of 144 participants aged 60–80 years participated. The primary outcome was subjective well-being, and the secondary outcome was sedentary behavior. The RCT aimed to evaluate the impact of yoga and light physical activity therapy on the health and well-being of older people over 60 years of age and to investigate side effects such as pain, mobility, sleep, memory, mood, stress, anxiety, depression, cardio-metabolic and inflammatory factors, as well as related sedentary behaviors [19]. Assessments were performed at zero weeks, after the intervention (13 weeks), and at follow-up (37 weeks). Participants had to attend twelve weeks of supervised classes for three months, each class lasting for about one hour, and they were also advised to practice at home. The physical activity styles of the participants were classical yoga and light exercise. They were followed for six months after the end of the last session. The yoga intervention included physical postures (asanas), breathing exercises, and moderate meditation [19]. Light exercise that was included was focusing on conventional stretching designed to improve mobility. Details of interventions are described elsewhere [19]. The instructors were qualified physical exercise tutors who had considerable experience teaching both yoga and other physical activities.

### 2.2. Study Participants and Data Collection Procedure

The participants in the qualitative study were the elderly population who participated in the RCT intervention. Of the 144 participants in the RCT, around 50 to 60 participants were contacted face to face for focus group discussions (FGDs); however, 30 to 36 were recruited purposively in each phase based on their availability and willingness to participate in the qualitative study.

A total of 18 focus group discussions (FGDs) were conducted—six FGDs for each phase of the RCT interventions (before, during, and after). Of the six FGD for each phase, three FGD included only male participants and three FGD only female participants. There were between five to six participants in each FGD. The FGDs were homogenous in terms of the gender of the participants; an equal number of males and females was chosen for the FGDs. The average age of the participants was 66 years, ranging from 65 to 70 years. Most of the males were retired, but some were engaged in business. The majority of the females were homemakers, and few were retired from their job.

The first, second, and last authors, who were fluent in the Hindi language, and have been trained in public health as well as qualitative research methods, facilitated the FGDs. They were also RCT investigators. Before conducting the FGDs, they established a good relationship with the participants to obtain in-depth information. In addition, the co-investigators of the study have medical, clinical, environmental, social, and nursing research backgrounds, all with a public health perspective.

A semi-structured open-ended guide was used for FGD. The guide was developed to discuss participants’ understanding of yoga/light exercise and their experience regarding the role of yoga/light exercise in the prevention of sedentary behavior. Basic demographic characteristics of the participants, date, and duration of the FGDs were also documented in field notes. The FGDs took place in the Ujjain Charitable Trust Hospital Seminar Hall, and during the FGDs, none of the non-participants were presented. We did not conduct any repeat interviews. No financial incentives were provided to the participants.

### 2.3. Data Management and Analysis

All FGDs were digitally recorded, transcribed, and translated into English. The average duration of the FGDs was 40 (range: 30–60) min. Both manifest and latent content analysis approaches were used for data analysis [19]. The results were generalized to an interpretive level; the facts specifically seen in the interview and the underlying context of the interview were interpreted. The inductive approach was followed for the analysis of data. The data from each phase, i.e., before, during, and after the intervention, were analyzed separately. The meaning units, i.e., parts of the original text that were relevant to the purpose of the study, were identified from the interview transcript. The meaning units were coded, and similar codes were grouped and collapsed into categories. In the final step of the analysis, patterns were identified and described both in-depth and in relation to each phase. Several steps were taken to ensure trustworthiness: memos were used to track the analysis process, and the analysis was repeated among the authors to conceptualize and interpret the findings.

### 2.4. Ethical Considerations

The study was approved by the Institutional ethics committee of R. D. Gardi Medical College, Ujjain (IEC Ref. no-09/2018-C). Participants were informed about RCT interventions and their participation in the qualitative study. Written informed consent was obtained from participants before their participation. They were assured of confidentiality and of their right to withdraw from the discussion whenever they wished, and that there would be no effect on their ongoing intervention in the event of any decline. Permission was obtained for the audio recording of the discussion.

## 3. Results

The findings regarding motivating and demotivating factors in various phases of intervention were presented in three categories: experience and perception of the effects of yoga/light exercise on sedentary behavior (1) before, (2) during, and (3) after intervention (Table 1). The findings were illustrated with quotations from the participants.

### 3.1. Category 1: Motivating and Demotivating Factors Regarding the Effects of Yoga/Light Exercise on Sedentary Behavior before Intervention

Factors that motivate the participants to practice yoga/light exercise before the interventions, as reported, were confidence in the benefits of yoga/light exercise, the ability to stay fit and safe, fear of ending up in bed, and professional guidance. Most participants agreed that yoga/light exercise had a positive effect on sedentary behavior. Most participants expressed the importance of physical activity for a healthier life and mentioned that they have learned a lot about the benefits that can be achieved when doing yoga/light exercise. They felt that a lifestyle with some kind of physical activity was necessary in old age.

“Yoga/light exercise enhances our immune power; breath regulation controls many things in our body. Physical activity will sustain your mobility and that is why yoga/light exercise is very necessary.” Before intervention, female participant.

Household work, commitments, and time management issues have been reported as significant demotivating factors for yoga/light exercise among the elderly. Many participants were discouraged or unable to participate in yoga/light exercise due to lack of personal space, inadequate awareness, and lack of encouragement from family members. Ignoring their health and lack of awareness of physical activity was another obstacle identified by many participants. Many of them found household tasks to be physical exercise, but few of them were concerned about safety due to age factors. Women participants were more worried about their household time management and family support compared to men.

“I have never had time; I am still occupied with homework. I could never practice either yoga or exercise because of too much stress, even though I wanted to. I’m taking care of my grandson. I can’t abandon him and come, so he doesn’t go anywhere. I’m overweight and I can’t do any sort of exercise. Even if I join your program, I will not be able to continue! I have a lot of questions about getting my family support.” Before intervention, female participant.

It was noted that despite the fact that some participants perceived barriers to joining yoga/light exercise, many elderly people were aware of the importance of fitness and showed a great deal of interest in such a program. They said they would like to take part in this program, but were unsure if they would be able to since it is carried out over a fairly long time. They were concerned about time management for housework and other involvement in the family and society; however, most of them stated that if they received family support, they would join the program. Some participants were not sure of the outcome of the program, while some expressed positive expectations of participating in the proposed yoga/light exercise program. They have a variety of reasons for participating in this program, including health benefits, desire to stay fit, boredom about their present situation and current monotonous work, and the search for some change.

“People are not compelled to take care of themselves unless they are confronted by any illness. I feel terribly lonely at home and my family is burdened; yoga has amazing effects; yoga has healed cancer, heart diseases. I have pain in my leg for a long time; hopefully after exercising at your middle, it will decrease.” Before intervention, male participant.

### 3.2. Category 2: Motivating and Demotivating Factors Regarding the Effects of Yoga/Light Exercise on Sedentary Behavior during Intervention

Participants highlighted a number of factors that encouraged them to participate in yoga/light exercise interventions, and many of them developed positive attitudes. The key motivating factors identified during the intervention phase were periodic health check-ups and consultations with physicians, and improvement in self-health status, which gradually helped them gain family support. Most of the participants said they were gaining more benefits and enjoying participating in yoga/light exercise classes under the guidance of a professional trainer without any payment or cost. According to the participants, the interesting way of training motivates them to attend the class regularly. They realized that, at no cost, they were being provided with more facilities to perform yoga/light exercise; this was not possible at home, and it encouraged them to motivate their family members to seek their support.

“Initially, I thought I might get hurt while doing yoga or exercise as the body grows weak with age. Carelessness is, in my opinion, the biggest reason. I was practicing in my home in the wrong way, with negative impact or no impact. We can’t practice at home like a class. I try it at home, but it wasn’t as enjoyable as it was in the classroom. We have to be correct in the classroom; we can do what we want at home. Therefore, I continued to enjoy the class and get more health benefits.” During intervention, male participant.

“I went to the Orthopedic, he advised me to join the yoga/light exercise class, he told me that medicines would not help. Usually we’re going to have to pay yoga/light exercise class, but I’m going to get the class free of charge.” During intervention, female participant.

During the program, the participants provided a class review. They highlighted the positive and negative changes they experienced in their health while participating in the program. According to their experience, they gained energy and enthusiasm in the morning and became active. Many of the participants explained that their body pain had reduced after practicing yoga/light exercise, and most of them thought that their daily routine had improved after they started practicing yoga/light exercise. From morning to night, in every aspect, whether eating or going out, everything improved, they felt good, and their digestive system remained healthy. They cited the mentorship, supervision, and attention that they received from the staff in the program, and the instructors encouraged them to attend regularly.

“Our own relatives don’t behave like the staff of the program do. We feel great when you show so much concern for our well-being; staff members have so much concern for us, even more than our family. They (instructors) pay attention to each and every participant and even hold their hands to clarify the procedure.” During intervention, male participant.

“We’re very happy to be part of this program, we’re going to be extremely happy if you continue to do this and continue to benefit people like this, yoga/light exercise can increase your life span when you gain control over your breathing, and you can increase your life span.” During intervention, female participant.

The majority of participants felt that the three-month duration of the program was insufficient, as evident from their requests for the continuation of classes. Many of them are pushing for the long-term continuation of the program. Participants and former program participants who experienced numerous positive changes during the classes also inspired their family members and others to participate.

“The members of my family encouraged me to attend classes. Even my neighbor was enrolled in this program before me, and I improved his health and pain, so I decided to keep going.” During intervention, female participant.

Most of the women involved felt that household work was a major challenge for time management. The other barrier that emerged was a lack of personal space, as many women feel shy about practicing yoga/light exercise in front of other family members. When the guest arrived, it was difficult to continue the exercise—they were responsible for providing the guests with refreshments. One barrier that came to light while talking to old adults before they participated in the program was that almost all females previously thought that they do not need to practice physical activity, as they already do household chores which provide a sufficient workout. Some participants quit at the beginning of the session, but were later advised by instructors and other staff members to continue to attend classes.

“I can’t get time at home, sometimes, whenever I get time to exercise, that’s why I’m saying the program shouldn’t be over. I’ve already had very good results, but I still want the program to continue forever.” During intervention, male participant.

### 3.3. Category 3: Motivating and Demotivating Factors Regarding the Effects of Yoga/Light Exercise on Sedentary Behavior after Intervention

Improved mobility; flexibility; reduced stiffness; joint pain; and mental benefits such as better concentration, self-confidence, stress relief, anger management, and better sleep quality were key motivating factors for the continuation of yoga/light exercise among the elderly after the intervention phase.

The participants discussed several reasons for them to continue the program. Many of them highlighted the number of benefits that their body experienced after completing yoga/light exercise, which included improving mobility and joint pain. According to the participants, exercise relaxes all of the muscles of the body that otherwise feel stiff, the body stays energetic, the appetite improves, the circulation of the blood improves, and the movement of the muscles relaxes. They perceived an improvement in their immune system. The participants also discussed the improvement in mental health benefits and the enhancement of well-being. They experienced better concentration, self-confidence, stress relief, anger management, and better sleep quality, and most of them have talked about continuing family support.

“The instructors pay attention to each participant and provide handhold support. My mind is very peaceful right now. I used to worry a lot earlier. In our lives, we had hopelessness, disinterest. But all negative emotions were gone. We used to get tired easily, and that fatigue haunted us mentally, I used to feel old, and my time is almost over, after yoga/light exercise, I feel like I’m young.” After intervention, male participant.

“An elixir that we have to take to be physically healthy and mentally calm. I can walk easily, and I feel healthy. I seem to have turned ten years younger. I mean, actually, I’m 75 years old and I feel like I’m 65 years old. We felt happy to meet people of our own age group in the classroom.” After intervention, male participant.

“I had hypersomnia, I just wanted to sleep and sleep. I’m feeling tired. Even after lunch, I don’t need to sleep. I couldn’t work before after I had a meal. For a while, I felt like I needed to rest. This is the biggest change in my life after practicing yoga/light exercise.” After intervention, female participant.

“I used to be a short-tempered person, but now I’ve learned to control my anger, even though I know I can’t get rid of it, but now I can control it.” After intervention, male participant.

“I am more than happy to join this program, and it is true that after I have participated, I feel extremely energetic in my body, and I have experienced an improvement in concentration, and I feel that programs like these should go on without ending.” After intervention, female participant.

Elderly participants further stated that attending the program breaks the monotony of their routine, that they have had the opportunity to exercise and meet other elderly people and discuss a lot of topics from politics to the environment, and that they can engage with a lot of people in the group. Many of the participants were unable to complete the full module or were unable to attend all classes. They cited many reasons, including transport, other household responsibilities, family pressures, and lack of proper time management. Some participants raised issues such as laziness, lack of training, and fear of getting hurt for not attending the full module.

“Morning hours are busy times for me and a lot of work at home. My son wasn’t happy that I was going to exercise in the morning, so I had to stop doing what I could do?” After intervention, female participant.

“Laziness is the most important reason for this. They’re always looking for excuses.” After intervention, male participant.

## 4. Discussion

This study provides an insight into how physical activity is perceived among the elderly before, during, and after intervention. It was noted that, before joining the interventions, the elderly had many demotivation factors for yoga/light exercise, which were essentially due to lack of awareness and a suitable environment, place, time, and coaching of yoga/light exercise trainers. After the intervention, however, almost all participants experienced the value of yoga/light exercise in managing their sedentary behavior; this was due to increased health literacy and the provision of proper guidance without any out-of-pocket expenditure. It emerged that yoga/light exercise were both seen as appropriate activities for older adults, as it could be practiced despite their older age. It has been reported that practicing yoga/light exercise has many benefits, such as increased flexibility and strength, pain relief, joint stiffness, chronic fatigue, and improved mental health and well-being.

The perceived benefits of yoga/light exercise after intervention in our study were improved strength and flexibility, better sleep quality, energy level, help with the joint problem, fatigue, and social behavior, which have also been reported in a similar study in India [20]. The findings of our study are supported by similar intervention studies, which validate that yoga/light exercise effectively improve pain management, strength, flexibility, and sleep [21,22,23,24,25]. Many participants in the yoga/light exercise classes also claimed to observe enhanced memory, self-confidence, feeling of happiness, and stress relief. The majority of the participants reported that meditation could balance the age-related decline in the cognitive domain of memory. However, some other domains, such as judgment, language, intuition, and the ability to learn, are reported to have an association with meditation in similar studies [26]. In the case of males, laziness and ignorance regarding personal health were revealed to be the biggest barrier to participation in any physical activity program, while in the case of females, household responsibilities, work, lack of time, and lack of knowledge were reported as the major barriers. Our findings concur with those reported by other studies [27]. Safety concerns and lack of professional instructors were other barriers identified in our study, and these findings were in line with other studies [28]. Many participants also reported house infrastructure as a barrier to practicing physical activity at home. In the case of women, lack of a personal room or place where they can practice without being disturbed or without feeling self-conscious was also reported as a barrier. Engaging in a yoga/light exercise program was preferred over self-practice in our study. Time management was another barrier identified among working males, as well as household responsibilities among females, which are in line with a similar study [29].

Another finding that serves as an obstacle is physical disabilities due to aging, such as fatigue, weakness, and body pain, but this obstacle also serves as a motivating force for those who wish to stay healthy. In addition, the severity of disease is a deciding factor for participants’ behavior. A report published [30] supports our finding. The study participants also stated that they thought about quitting the program when they experienced pain at the beginning of their session, but they were advised by their instructor to continue practicing. The instructor plays a crucial role in helping the participant not only in performing the exercises correctly but also in motivating the participant to practice regularly. Instructor quality was reported as a perceived barrier and/or facilitator in other studies [30,31,32], highlighting the need for positive and encouraging words from trainers. Instructors can play the role of a psychological counselor who can make the participants relieve their stress and depression with encouraging words. According to our findings, other driving factors are family support, doctor’s advice, and motivation from others who have benefited and changed their sedentary behavior after practicing yoga/light exercise. Personnel activity plays a very important role in the case of inclusion in the system.

The effectiveness of yoga practice as a therapeutic technique has rarely been addressed. Similarly, discussing the impact of yoga on stress is essential. Hence, both clinical and non-clinical experts have suggested the use of yoga as a therapeutic technique [33,34]. Mental health professionals in the yoga community have observed a significant reduction in work-related stress (and a significant increase in stress adaptation) and an increase in autonomic nerve function in a 12-week weekly yoga program. Physicians, managers, and educators could give yoga classes as a course [35].

This study illustrated the views of older adults who had no prior experience with yoga/light exercise. This study identified multiple barriers to participation in the yoga/light exercise program and home practice of physical activity. The instructors were the same for all of the groups, and all of the participants in the group belonged to the same party. Participants had the same components practiced in their respective groups. The study’s strength is that codes and categories were continuously discussed between the research team until a consensus was reached. The limitation of this study is that convenient sampling was conducted.

## 5. Conclusions

This study explores the feasibility of practicing yoga/light exercise and its effect on the sedentary behavior of the elderly population. They were recognized to have undergone changes in their physical and emotional health and well-being by consistently practicing yoga/light exercise. The main driving factors were periodic health check-ups, enhanced self-esteem status, and a qualified trainer’s encouragement without any cost. Nevertheless, laziness between males and household responsibility among females were seen as major barriers to involvement in any program and practice of physical exercise at home. The study concludes that these interventions should be encouraged in the community to use physical exercise as a method to better control the physical and social effects of aging. These insights may help instructors, researchers, yoga studios, fitness centers, program developers, and designers.

## Figures and Tables

**Table 1:** Motivating and demotivating factors on the effects of yoga/light exercise on sedentary behavior before, during, and after the intervention.

	Before Intervention	During Intervention	After Intervention
**Motivating factors**	Belief in the benefits of yoga/light exercise Desire to stay fit and healthy Fear of ending up bed-bound Professional guidance	Interesting classes Free health check-up Supervised classes Family support	Improvement in mobility, flexibility, stiffness, and joint pain Mental health benefits Better concentration and self-confidence Relief from stress Better sleep quality Escape from monotony
**Demotivating factors**	Household work Problems with time management Lack of personal space Considering household chores as a physical workout Lack of knowledge Safety concerns due to age No support from family	Household work Problems with time management Difficulty in performing yoga and mild exercise postures	Household work Time management
